# Age predicts Alzheimer's in Down syndrome better than MRI, plasma, or cognition

**DOI:** 10.1002/alz.71661

**Published:** 2026-07-14

**Authors:** James T. Kennedy, Julie K. Wisch, Benjamin L. Handen, Sigan Hartley, Adam M. Brickman, Joseph H. Lee, Sharon Krinsky‐McHale, Florence Lai, Herminia Rosas, Elizabeth Head, Bradley Christian, Shahid Zaman, Ira T. Lott, Christy Hom, Lauren T. Ptomey, Jeffrey M. Burns, Anne Cohen, Dana Tudorascu, Charles Laymon, Patrick Lao, Melissa Petersen, Frederick Schmitt, Beau M. Ances

**Affiliations:** ^1^ Department of Neurology Washington University School of Medicine in St. Louis St. Louis Missouri USA; ^2^ Department of Psychiatry University of Pittsburgh Pittsburgh Pennsylvania USA; ^3^ Waisman Center University of Wisconsin‐Madison Madison Wisconsin USA; ^4^ Department of Human Development & Family Studies University of Wisconsin‐Madison Madison Wisconsin USA; ^5^ Department of Neurology Columbia University New York New York USA; ^6^ Gertrude H. Sergievsky Center and Taub Institute for Research on Alzheimer's Disease and the Aging Brain Columbia University New York New York USA; ^7^ Department of Epidemiology Columbia University Irving Medical Center New York New York USA; ^8^ Department of Psychology New York State Institute for Basic Research in Developmental Disabilities New York New York USA; ^9^ Department of Neurology Harvard Medical School, Massachusetts General Hospital Charlestown Massachusetts USA; ^10^ Department of Radiology Harvard Medical School, Massachusetts General Hospital Charlestown Massachusetts USA; ^11^ Department of Neurobiology and Behavior University of California Irvine California USA; ^12^ Department of Pathology University of California Irvine California USA; ^13^ Department of Medical Physics University of Wisconsin‐Madison Madison Wisconsin USA; ^14^ Cambridge Intellectual and Developmental Disabilities Research Group University of Cambridge Cambridge UK; ^15^ Department of Pediatrics University of California Irvine School of Medicine Irvine California USA; ^16^ Psychiatry & Human Behavior University of California Irvine California USA; ^17^ Cardiovascular Research Institute, Department of Internal Medicine University of Kansas Medical Center Kansas City Kansas USA; ^18^ University of Kansas Alzheimer's Disease Center Kansas City Kansas USA; ^19^ Department of Radiology University of Pittsburgh Pittsburgh Pennsylvania USA; ^20^ Institute for Translational Research University of North Texas Health Science Center Fort Worth Texas USA; ^21^ Alzheimer's Disease Research Center University of Kentucky Lexington Kentucky USA; ^22^ Sanders Brown Center on Aging University of Kentucky Lexington Kentucky USA; ^23^ Department of Neurology University of Kentucky Lexington Kentucky USA

**Keywords:** Alzheimer's disease, biomarkers, cognition, diagnostics, Down syndrome

## Abstract

**INTRODUCTION**: Alzheimer's disease (AD) dementia in Down syndrome (DS) occurs at predictable ages. It is unclear whether age can differentiate across AD stages (amyloid positivity, tau positivity, mild cognitive impairment [MCI], dementia).

**METHODS**: Using data from the Alzheimer's Biomarker Consortium–Down Syndrome, we analyzed how well age differentiated stage using receiver operating characteristic curves. We compared areas under the curve (AUC) for age to AUCs for imaging, biofluid, cognitive, motor, and behavioral variables.

**RESULTS**: Sample varied by stage and variable. Up to 148 variables and 461 participants were analyzed. Age effectively differentiated amyloid positivity, tau positivity, and MCI (AUCs > 0.85) but poorly discriminated MCI from dementia (0.588). No variable was better than age in distinguishing stages, except for MCI/dementia.

**DISCUSSION**: Our results show that age alone is effective at staging DS AD. Age is the most reliable correlate of amyloid, tau status, and cognitive impairment in DS and could screen for future clinical trials.

## Highlights

- Age alone was comparable to or exceeded the discriminative value of AD tests in DS.
- Combining age and memory increased discrimination for AD clinical impairment.
- Amyloid and tau accumulation begins around 42 years old in DS.
- AD impairment can begin as early as 45 years old in DS.
- Memory tests were the only effective measure when discriminating MCI and dementia.

## 1 BACKGROUND

People with Down syndrome (DS) have a 90% chance of developing Alzheimer's disease (AD) due to the triplication of the amyloid precursor protein (APP) gene on chromosome 21 leading to an overexpression of amyloid beta (Aβ).^1^, ^2^ In general, AD in DS has a typical progression characterized by the accumulation of amyloid beta plaques in the brain, followed by the accumulation of tau neurofibrillary tangles, leading to neurodegeneration and cognitive impairment.^3^ This process may be accelerated in DS^4^ relative to sporadic/late‐onset AD.^5^ Amyloid typically accumulates in DS between the mid‐30s to early 40s,^6^ with tau pathology emerging within 2 to 4 years^7^, ^8^ and cognitive decline occurring in the early/mid‐50s.^1^, ^2^, ^9^, ^10^ The stereotypical pattern and timing of AD in individuals with DS make AD staging more predictable compared to late‐onset AD.^1^

AD staging in DS can be defined with biological and cognitive measures, but they are not without drawbacks.^11^, ^12^ Amyloid and tau positron emission tomography (PET) are the gold standard for identifying the accumulation of AD pathology but are expensive and require access to PET scanners.^13^, ^14^ Similarly, premorbid differences in cerebrospinal fluid (CSF) and plasma amyloid levels between DS and late‐onset AD^15^ make diagnostic thresholds derived from the latter questionable for the former. Cognitive decline is usually assessed through cognitive task performance and informant interviews about changes in memory, adaptive skills, behavior, and other areas. These assessments can be influenced by the varying levels of premorbid intellectual impairment in DS and intra‐individual performance fluctuations unrelated to AD pathology.^15^ Due to barriers in accessing gold‐standard methods for identifying AD stages, alternative approaches need to be validated for DS. The predictable timing of AD stages in DS makes age a viable alternative for screening in individuals at high risk for AD. This observation suggests that age could be a useful alternative for staging AD in DS, especially given the limitations in the availability of other measures. Additionally, age should be considered in comparison to other existing markers used for identifying AD stages in DS.

While previous research pointed to a fairly predictable time course for AD progression in DS,^1^, ^6^, ^7^, ^16^ the effectiveness of age as a diagnostic measure is uncertain, and its utility compared to existing measures remains unclear. Currently, biomarker and cognitive assessments are commonly used to identify disease stages, each with varying degrees of accuracy,^15^ but it remains unclear whether existing measures and thresholds are better than age or whether adding age to these diagnostics would improve accuracy. Given that age is a major risk factor for dementia, we hypothesized that age might be a stronger predictor of AD stage in DS than existing biomarkers. We evaluated this hypothesis using data from the Alzheimer's Biomarker Consortium – Down Syndrome (ABC‐DS).

## 2 METHODS

### 2.1 Study design

In this retrospective cohort study, data from the baseline session of the longitudinal multisite ABC‐DS study (consisting of nine sites across the United States and United Kingdom) collected between 2015 and 2025 were analyzed to examine how accurately age discriminated AD stage relative to known AD measures. ABC‐DS was initially two separate groups, the Neurodegeneration in Aging Down Syndrome (NIAD) study and the Alzheimer's Disease in Down Syndrome (ADDS) study, which were combined in 2020. All procedures were approved by the Institutional Review Boards (IRBs) of each site enrolled in the study.

### 2.2 Participants

Participants included people with DS recruited by ABC‐DS, NIAD, or ADDS. All studies enroll participants with DS and sibling controls who are at least 25 years old and who have an IQ of at least 30. People with a diagnosis of dementia were initially enrolled, but only cognitively stable individuals were enrolled after combining groups in 2020, though some initially stable individuals were diagnosed with dementia after enrollment. Other medical conditions that would interfere with cognitive assessment resulted in study exclusion. Participant age ranged from 25 to 70 years old. Medical records were reviewed, and if status was uncertain, participants underwent karyotype testing to confirm DS and type (full trisomy, mosaicism, or translocation). All participants had their apolipoprotein (*APOE*) ε4 carrier status established using a KASP assay (LGC Genomics [Beverly, MA, USA]), with the presence of at least one ε4 allele considered *APOE* ε4 positive. A total of 471 participants were included in these analyses, though the number of participants in each analysis varied as not all participants had usable data for every measure. Written informed consent and/or assent was obtained from all participants or, if individuals were incapable of providing consent, from their legally authorized representatives. All procedures were approved by the IRBs of the ABC‐DS member sites and were performed in accordance with the ethical guidelines in the 1964 Declaration of Helsinki and its amendments.

**RESEARCH IN CONTEXT**

1. **Systematic review**: We searched PubMed for journal articles examining the accuracy of different measures used to stage AD in DS through July 2025. Studies have identified the discriminative abilities of biomarkers and cognitive measures when examining disease stage but have not either examined their discriminative ability relative to age or compared age to a limited set of measures for a single stage comparison.
2. **Interpretation**: This study found that age either outperformed or matched the discriminative ability of all measures tested (imaging, biofluid, and cognitive measures) until differentiating MCI from dementia, where only memory tests discriminated stage.
3. **Future directions**: The highly predictable nature of DS AD provides an opportunity for very early intervention and as a screening tool for clinical trials. There are also factors (e.g., degree of premorbid intellectual impairment) that may affect how well variables differentiate stage.

### 2.3 Procedures

#### 2.3.1 Measures

We considered nearly every continuous variable (*n* = 148) tracked by ABC‐DS. Analyses were limited to each participant's first session of data to avoid introducing possible practice effects for cognitive tests. For tests that required a repeated number of trials, summed raw test values rather than individual trial results were used. A full list and description of variables is available in the Supporting Information and includes age, measures of cognition, motor function, psychopathology, social ability, biofluids (CSF and plasma markers of AD and inflammation) and imaging (regional magnetic resonance imaging [MRI] brain volume, cortical PET amyloid, tau PET summary region) biomarkers. We also used summary values from several tests, such as combined scores of cognitive scales instead of separate short‐ and long‐term memory measures.

##### Biomarkers

###### Fluid biomarkers

Fluid biomarkers included nine CSF and 28 plasma measures associated with AD. CSF was analyzed with a LUMIPULSE G1200 (Fujirebio, Malvern, PA, USA) immunoassay, a Quidvel MicroVue Bone plate‐based assay, or a Single Molecule Counting Singulex Erenna system. Plasma was measured on a Meso Scale Discovery (MSD) platform or a Quanterix Single Molecule Array (Simoa) platform, the latter run on the HD‐1 imager. A full list of CSF and plasma markers, how they were measured, and their clinical relevance can be found in Table S1.

###### Imaging biomarkers

Imaging markers consisted of regional brain volume derived from MRI and amyloid and tau PET. All MRI scans were collected on a 3T scanner and processed through FreeSurfer version 5.3 or 7.3^16^ to obtain regional brain volume estimates. Bilateral brain structure volumes were summed and corrected for intracranial volume.^17^ This resulted in 34 cortical and 10 subcortical gray matter regions. For PET, participants were administered [11C]‐Pittsburgh compound B (PiB) or [18F]‐AV45 (florbetapir) to identify amyloid plaque level and [18F]‐flortaucipir to measure tau accumulation. Amyloid PET data were collected for 50 to 70 min after injection for both tracers, and tau PET data were collected 80 to 100 min after injection. Both were processed through a PET Unified Pipeline (PUP)^18^ that aligns PET scans to FreeSurfer regions, applies partial volume correction using a regional spread function,^19^ and calculates standardized uptake value ratios (SUVRs) using the whole cerebellum as a reference region. A summary of amyloid from select regions was used to generate a summary value and converted into Centiloid values^20^ to harmonize across amyloid tracers. A cortical summary measure^21^ was used for tau.

##### Participant performance measures

Measures derived from participant performance or questionnaires covered mental status, memory, visual spatial ability, motor ability, and language skills.^22^, ^23^ Overall cognition was measured through the Down Syndrome Mental Status Examination (DSMSE),^24^ which examines performance in multiple cognitive domains, including memory, language, motor ability, knowledge, and visuospatial ability. Total and component tests were analyzed in the DSMSE. The Rapid Assessment for Developmental Disabilities^25^ hand movement and gesture imitation tests were used to assess motor planning and control. Executive processing and speed were measured using the Cats and Dogs Stroop task^26^ and the Purdue Pegboard.^27^ In the Cats and Dogs Stroop test, participants were asked to name the animal in a photo for naming trials and say the opposite animal in the switching trial. In the Purdue Pegboard, participants have 30 s to pick up and place nails into pegs on a pegboard one at a time, tested on both hands. Language and cognition were measured using a Verbal Fluency test,^28^ where a participant is asked to generate a list of words in a specific category in 20 s. Short‐term memory was measured using the modified Cued Recall Test (mCRT)^29^ in which participants first learn a list of words by being shown pictures of 12 items and given a category cue (e.g., fruit for picture of grapes). They are then asked to freely recall the words and given the category hint if they fail to recall them (21% of the mCRT data was omitted due to version compatibility issues). Free, cued, intrusions (non‐list words in a free or cued response), and total score were used for mCRT. Visuomotor and visuospatial ability was measured in the Beery‐Buktenica Developmental Test of Visual Motor Integration (VMI),^30^ the Wechsler Intelligence Scale for Children, Fourth Edition's Block Design,^31^ and the Haxby extension to the Block Design task (block tasks measured separately and together).^24^ In the VMI, participants have to copy 24 geometric figures. The Block Design tasks have participants recreate a pattern using partially or fully colored blocks. Gait was measured by the Tinetti Assessment Tool.^32^

##### Informant measures

Multiple tests measured participant ability based on caregiver or informant report. Mental status was measured by the Dementia Questionnaire for People with Learning Disabilities (DLD)^33^ and the National Task Group – Early Detection Screen for Dementia (NTG‐EDSD).^34^ The DLD tracks functioning in eight cognitive or social domains while the NTG‐EDSD tracks changes in adaptive daily living, ambulation, behavior, communication, memory, and sleep. The Reiss Screen for Maladaptive Behavior^35^ measures maladaptive behaviors (e.g., depressed mood, disruptive behavior) across seven domains, as well as a total score.

#### 2.3.2 Clinical assessment

A clinical case consensus approach was used to determine AD clinical status.^23^ All available cognitive and informant‐reported measure scores were considered along with medical and psychiatric histories and clinical lab values. Raters were blind to biomarker results. Participants were placed in one of the following statuses: cognitively stable, mild cognitive impairment (MCI), AD dementia, or status uncertain if a secondary cause (e.g., medical, major life event) could have been responsible for cognitive change, if the participant did not engage with the tests, or if baseline performance was too low for the tests to detect meaningful change. The MCI and dementia groups were combined to form a cognitively impaired group (MCI+dementia).

#### 2.3.3 Stages of pathology

The discriminative ability of age was tested for different stages of AD. AD biomarker stages considered whether a participant was amyloid negative versus positive or tau negative versus positive. The clinical status stages considered included cognitively stable versus MCI, MCI versus AD dementia, and cognitively stable versus impaired (the combined MCI and AD dementia groups). Amyloid positivity was defined as ≥18 Centiloids.^8^ Tau positivity was defined as mean SUVR ≥ 1.22 in tau signature regions.^21^ Amyloid and tau PET variables were omitted from their respective stage comparisons as these values were used to define groups. The cognitive measures used when identifying dementia status were included in cognitive stage analyses, introducing some circularity. Only variables that were obtained in more than 10 participants in each of the stages were evaluated. The total number of participants in each stage for each variable can be found in the Supporting Information.

### 2.4 Statistics

#### 2.4.1 Demographics

Demographic differences between AD stages were compared with *t*‐tests for continuous measures and chi‐squared tests for categorical variables. Analyses were based on the entire sample. Measures compared included age, centiloid, tau, percent male, percent *APOE* ε4 positive, and karyotype.

#### 2.4.2 AD clinical status differentiation

The measures that were most strongly associated with stage were determined using receiver operating characteristic (ROC) curve analyses. ROC curve analyses compare the sensitivity (true positive/(true positives + false negatives)) relative to the specificity (true negative/(true negatives + false positives)) of every threshold across a continuous measure when differentiating a binary grouping variable, creating a curve that captures a portion of a square. The area of the square under the curve (AUC) describes how well the measure can differentiate groups, with an AUC of 0.5 meaning the measure separates groups randomly while an AUC of 1 means the measure can perfectly identify group membership. An AUC above 0.7 is considered acceptable, above 0.8 excellent, and above 0.9 outstanding.^36^ The threshold that best differentiated groups in ROC curve analyses was determined by the measure's Youden Index, which identifies the threshold with the best balance of sensitivity and specificity by taking the point on the curve farthest from the identity line.^37^ All analyses were done in R version 4.4.2. AUC and threshold confidence intervals were calculated using the pROC package.^38^

#### 2.4.3 Comparison of group differentiation ability

The ability to differentiate groups based on all variables was compared to their differentiation based on age. Analyses for each variable were limited to participants with both age and the variable of interest so that differences in sample could not influence results. AUCs, group defining thresholds, and their 95% confidence intervals (based on 2000 bootstraps) were calculated for all variables. ROC curves were compared using DeLong's test for difference in AUC^39^ use pROC's roc.test function. Multiple‐comparisons correction was applied across all variables and separately for each variable in a common set of analyses (e.g., all plasma variable corrected in one set, all DLD measures corrected in a separate set), with summary measures treated separately from their subcomponents due to non‐independence of the variables. This multiple correction approach was applied to the results from DeLong's test using a false discovery rate (FDR) correction where corrected values of *p* ≤ 0.05 were considered significant and *p* values between 0.05 and 0.1 were considered trends. Post hoc analyses examined age thresholds for amyloid and tau PET positivity using only participants with both scans to check whether differences in sample composition influenced age threshold estimates for these variables.

#### 2.4.4 Combining age with a second variable

We tested whether the discriminative ability of a variable could be increased by combining it with age. We performed a logistic regression on each grouping variable using the following formula: Group ∼ Age + Variable and Group ∼ Age + Variable + Age × Variable. Then we used the individual's predicted value from the models in ROC curve analyses. AUCs derived from the predicted values of these logistic regressions were compared against the AUCs from the individual age and variable AUCs using Delong tests. We did not withhold a subgroup of participants from the logistic regressions when testing ROCs due to the small sample size of some variables for some group comparisons. While this kept the sample constant across analyses, it might also have inflated AUCs as the same participants were used when defining and testing the logistic model. Potential heterogeneity in sample composition may bias results (e.g., more impaired participants may not have had usable results from more demanding tasks). The approach where variables were allowed to interact was compared to an approach where the variables were additive.

#### 2.4.5 Influence of *APOE* ε4

We further tested whether group differentiation could be increased by accounting for *APOE* ε4 carriership. Group membership was predicted from a logistic regression that included ε4 carriership and age, a secondary variable, their addition, or their interaction. ROC curve analyses were performed using the predicted values from these four models. AUCs from the ε4 models were compared to the AUCs from the original models where ε4 was not included using DeLong tests.

## 3 RESULTS

Participant number varied by measure and stages being compared, with as few as 36 participants for some CSF measures to as many as 461 in DLD and psychopathology (both in the comparison of cognitively stable to impaired, see Tables S2 to S5 for stage‐specific *N*s). The mean (standard deviation) sample size across analyses was 235.4 (97.7) for cognitively stable participants, 67 (33.4) for impaired, 38.3 (15.9) for MCI, 34.7 (14.3) for dementia, 124.4 (22.9) for amyloid negative, 87.1 (13.7) for amyloid positive, 115.6 (16) for tau negative, and 27 (4.1) for tau positive. We were able to evaluate all biomarkers considered in comparisons between cognitively stable and impaired participants. In all other comparisons (cognitively stable vs. MCI, cognitively stable vs. AD dementia, MCI vs. AD dementia), we were unable to evaluate classification of CSF‐derived measures due to insufficient samples. The comparison between MCI and AD dementia included the fewest biomarkers (*N* = 131).

Participant age increased with pathological stage progression. Amyloid‐negative participant age on average was 36.4 (6.5), amyloid positive 49.7 (6.4), tau negative 36.4 (6.7), tau positive 47.9 (6.5), cognitively stable 40.5 (8.8), MCI 52.3 (6.2), and dementia 54.1 (5.6). All group differences in age between stages were significant (*p* < 0.001) except when comparing MCI and dementia (*p* = 0.11). Centiloid likewise was higher with certain stages, with a Centiloid of 2.8 (5.8) for amyloid negative, 64 (33.9) for amyloid positive, 6.4 (11.9) for tau negative, 58.2 (47.9) for tau positive, 18.1 (28.4) for cognitively stable, 59.8 (36.9) for MCI, and 97.6 (36.8) for dementia. All stage comparisons were significant (*p* = 0.006 for MCI vs. AD dementia, all other *p* < 0.001). Tau also increased with each progressive stage. Amyloid‐negative participants had tau SUVRs of 1.06 (0.11), amyloid positive 1.61 (0.8), tau negative 1.04 (0.08), tau positive 1.82 (0.81), cognitively stable 1.12 (0.35), MCI 1.81 (0.75), and dementia 2.08 (1.24). This increase was significant across all stages except for the comparison between MCI and AD dementia (*p* = 0.71). Gender distribution was similar between groups. Impaired participants were more likely to carry an *APOE* ε4 allele than those classified as cognitively stable (*p* = 0.005). The proportion of participants with full trisomy 21 was similar across groups. The full demographics per group can be found in Table 1.

**TABLE 1:** Sample sizes and demographic variables for AUC analyses across variables.

	AB‐	AB+	Tau‐	Tau+	Stable	Impaired	MCI	Dementia
N range	37–149	43–101	66–134	15–33	22–353	12–108	14–56	17–52
N mean (SD)	124.4 (22.9)	87.1 (13.7)	115.6 (16)	27 (4.1)	235.4 (97.7)	67 (33.4)	38.3 (15.9)	34.8 (14.3)
Age mean (SD)	36.4 (6.5)	49.7 (6.4)*	36.4 (6.7)	47.9 (6.5)*	40.5 (8.8)	53.2 (6)*	52.3 (6.2)*	54.1 (5.6)*
Male%	54	64	57	55	53	57	66	48
*APOE* ε4%	19	23	21	18	21	25*	32	38
Tri/Trns/Mos/Unk%	90/6/3/1	93/2/4/1	90/7/2/1	97/0/3/0	90/5/3/2	84/3/6/6	91/2/5/2	77/4/8/12
Centiloid mean (SD)	2.8 (5.8)	64 (33.9)*	6.4 (11.9)	58.2 (47.9)*	18.1 (28.4)	77.6 (42.3)*	59.8 (36.9)*	97.6 (39.8)*
Tau SUVR mean (SD)	1.06 (0.11)	1.61 (0.8)*	1.04 (0.08)	1.82 (0.81)*	1.12 (0.35)	1.9 (0.89)*	1.81 (0.75)*	2.08 (1.24)

*Note*: Sample size varied by AD stage and measure. Demographic variable estimates and group differences are based on all participants with age. Continuous variables are expressed as the mean (standard deviation), categorical variables as a percentage. Asterisk denotes significantly different from the previous stage.

Abbreviations: AB, amyloid beta; *APOE* ε4, percentage with at least one ε4 allele; MCI, mild cognitive impairment; Mos, mosaicism; SUVR, standardized uptake value ratio; Tri, trisomy; Trns, translocation; Unk, unknown.

Age classified stage of AD biomarkers and clinical status at levels of AUC > 0.85 except for when differentiating MCI and AD dementia. Differentiating amyloid negative from positive had the highest AUC (0.920) and occurred at age 41.5. The age of 40.5 best discriminated tau status with an AUC of 0.883. Cognitively stable and MCI group discrimination had the second lowest AUC at 0.851, with an age of 44.5 separating groups. The age that best separated Cognitively stable and impaired was also 44.5 and had a slightly better AUC (0.870). MCI and AD dementia differentiation had a poor AUC at 0.588, with the age of 50.5 separating groups. The age that best identified tau status preceding the age identifying amyloid status was unexpected, though the age confidence intervals of tau (95% CI: 38.5 to 47) and amyloid (40.5 to 46.5) spanned several years and largely overlapped. Post hoc analyses limiting participants to only those who had both amyloid and tau PET scans found the same age threshold (40.5) for both PET measures with similar AUCs observed (0.919 for amyloid and 0.885 for tau), suggesting sample composition may have contributed to the later amyloid age threshold. AUCs dropped slightly with each progressive stage, though only the AUC for MCI versus AD dementia was significantly lower than the other AUCs (all *p* < 0.001).

**FIGURE 1:**
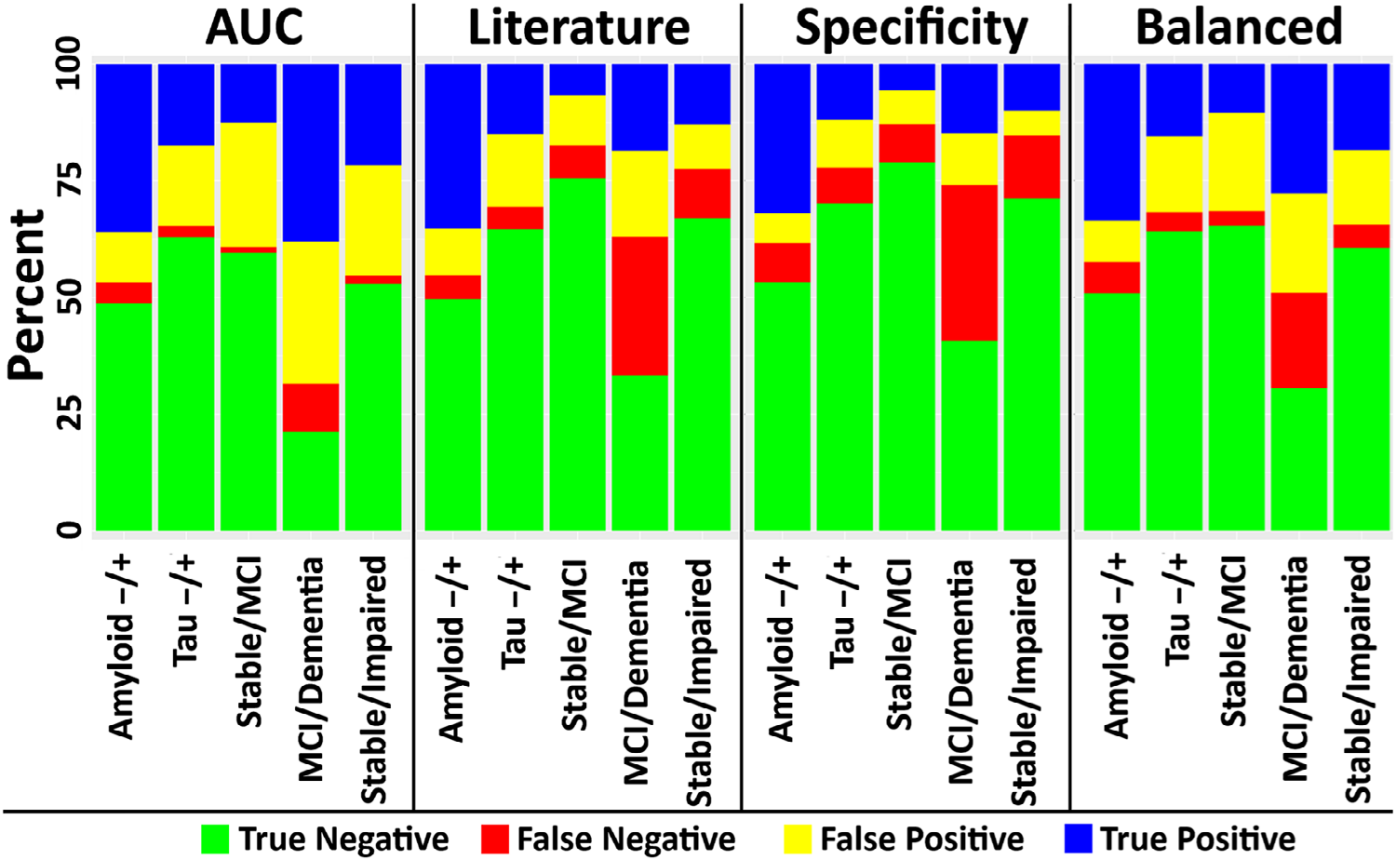
Confusion matrices for different thresholds. Proportional confusion matrices for each stage of AD progression based on receiver operator characteristic (ROC) curves, literature‐based age thresholds, a specificity‐driven threshold, and a threshold that balanced sensitivity and specificity. ROC thresholds tend to minimize false negatives (red columns) at the cost of greater false positives (yellow), while preserving true positives (blue) at the cost of true negatives (green). Sample size varies by comparison.

The thresholds for cognitive stages were lower than previously published.^1^, ^9^, ^10^ When distinguishing cognitively stable from MCI or impaired, we found that 44.5 years old best differentiated groups, while previous research placed the age at clinical symptoms in the early to mid‐50s.^1^, ^9^ The AUC prioritized sensitivity (0.926 for cognitively stable/impaired and 0.911 for cognitively stable/MCI), at the cost of specificity (0.691 for both). The ROC curve analyses consistently favored sensitivity across all stages compared. The gap between sensitivity and specificity was largest for the cognitive measures. Using the literature‐based 52.5 years old as the age at cognitive symptoms results in the opposite pattern (sensitivities of 0.556 for cognitively stable/impaired and 0.482 for cognitively stable/MCI, specificities of 0.875 for both). To identify age thresholds using an approach that favored specificity over sensitivity, we identified the age threshold at the specificity that most closely matched the ideal sensitivity from the ROC curve analyses. This resulted in a cognitive impairment age threshold of 54.5 (sensitivity = 0.426, specificity 0.929), later than most literature estimates.^1^, ^6^, ^9^ We also identified the age threshold where sensitivity and specificity were most similar, finding an age at impairment of 48.5 with a sensitivity of 0.787 and a specificity of 0.790. The effect of these different approaches to identifying thresholds is demonstrated in Figure 1, where the proportional confusion matrices for the AUC, literature‐based, specificity favoring, and sensitivity–specificity balancing thresholds are plotted for each stage comparison. The ROC‐derived thresholds consistently minimize false negatives at the expense of a largely balanced number of true and false positives (except for Centiloid, where true positives outweigh false). Sample sizes, AUC for the ROC curve analyses, and thresholds, sensitivities, specificities, and percent of sample positive before and after each threshold can be found in Table 2. Note that the percent positive after each threshold in no way implies that the inverse percentage of participants will not develop this stage of AD pathology, only that they have not reached the threshold at the age specified.

**TABLE 2:** Age thresholds for AD stage sample size, ROC statistics, thresholds, and 95% confidence intervals for AUC, literature‐derived thresholds, thresholds from when specificity matches the sensitivity of the ROC analyses (specificity), and thresholds where the difference between sensitivity and specificity was minimized (balanced).

	Stage comparison	N A	N B	AUC	AUC CI	Thresh	Thresh CI	Sensitivity	Specificity	% + <	% + >
ROC	AB‐/AB+	149	101	0.920	0.887–0.954	41.5	40.5–46.5	0.891	0.819	8.3	76.9
	Tau‐/Tau+	134	33	0.883	0.824–0.942	40.5	38.5–47	0.879	0.784	3.7	50.0
	Stable/impaired	353	108	0.870	0.838–0.902	44.5	42.5–48.5	0.926	0.691	3.2	47.8
	Stable/MCI	353	56	0.851	0.81–0.893	44.5	42.5–48.5	0.911	0.691	2.0	31.9
	MCI/dementia	56	52	0.588	0.48–0.696	50.5	46.5–57.5	0.788	0.411	32.4	55.4
Literature	AB‐/AB+	149	101	—	—	42	—	0.891	0.819	8.3	76.9
	Tau‐/Tau+	134	33	—	—	42.5	—	0.758	0.806	6.9	49.0
	Stable/impaired	353	108	—	—	52.5	—	0.556	0.875	13.4	57.7
	Stable/MCI	353	56	—	—	52.5	—	0.482	0.875	8.6	38.0
	MCI/dementia	56	52	—	—	55	—	0.462	0.607	45.2	52.2
Specificity	AB‐/AB+	149	101	—	—	44.5	—	0.792	0.893	13.6	83.3
	Tau‐/Tau+	134	33	—	—	45.5	—	0.606	0.873	10.0	54.1
	Stable/impaired	353	108	—	—	54.5	—	0.426	0.929	15.9	64.8
	Stable/MCI	353	56	—	—	53.5	—	0.411	0.915	9.3	43.4
	MCI/dementia	56	52	—	—	56.5	—	0.308	0.786	45.0	57.1
Balanced	AB‐/AB+	149	101	—	—	43.5	—	0.832	0.852	11.8	79.2
	Tau‐/Tau+	134	33	—	—	41.5	—	0.788	0.799	6.1	49.1
	Stable/impaired	353	108	—	—	48.5	—	0.787	0.790	7.6	53.5
	Stable/MCI	353	56	—	—	47.5	—	0.768	0.756	4.6	33.3
	MCI/dementia	56	52	—	—	53.5	—	0.577	0.589	40.0	56.6

Abbreviations: AB, amyloid beta; CI, confidence interval; MCI, mild cognitive impairment; A, first stage listed; B, second stage listed; Thresh, age threshold; % + </> ‐ percentage of participants positive for the second group listed before/after the threshold.

**FIGURE 2:**
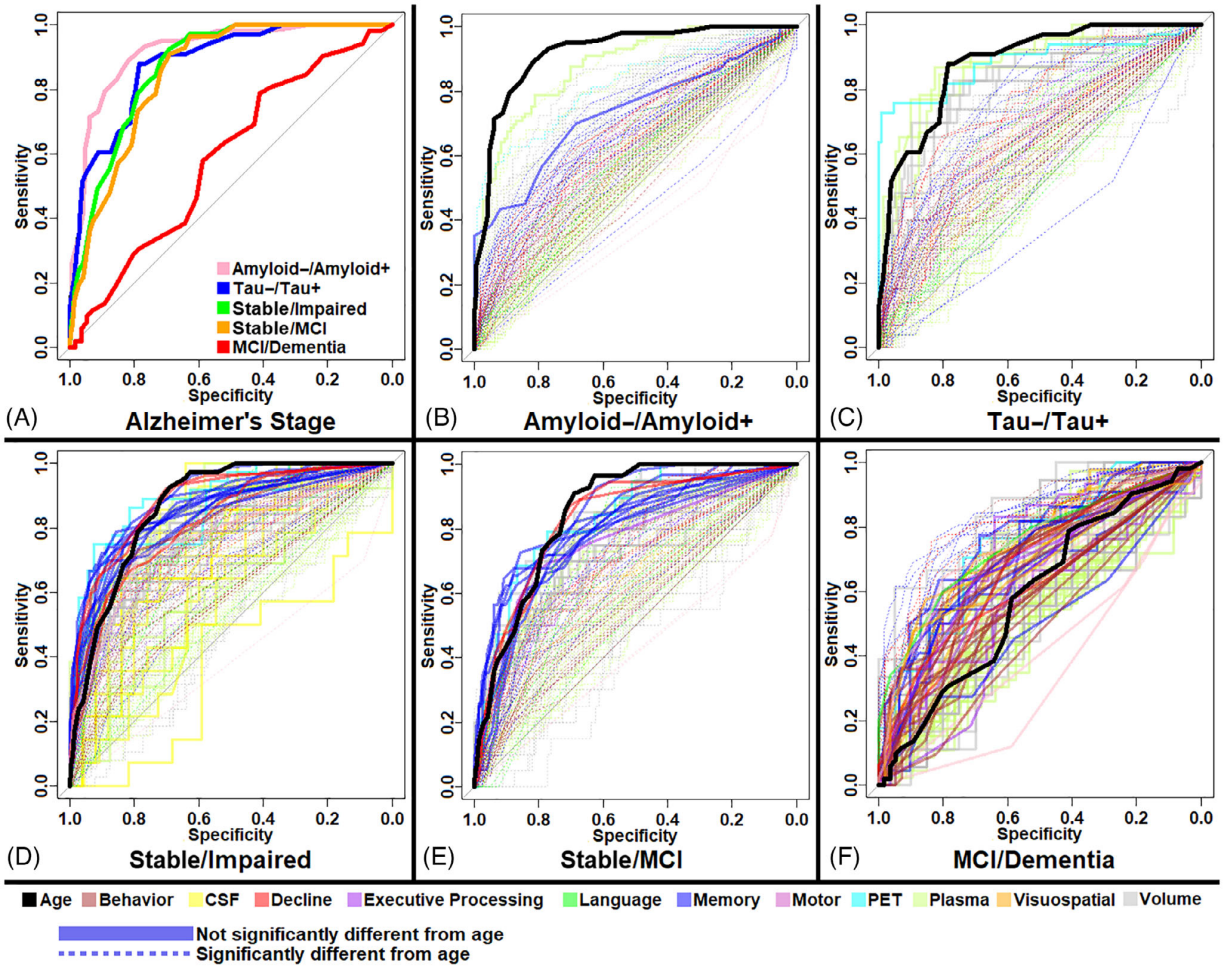
ROC plots comparing the discriminative ability of age and other variables by AD stage. Receiver operator characteristic (ROC) curve plots for all analyses, color coded by stage (A) and by type of measure (B–F). Panel A shows the age ROC curves for each stage of AD progression as different colors, while panel B–F show age in black and all other variables grouped by the type of measure (e.g., plasma, memory) used. Wide solid lines in (B)–(F) represent measures whose AUC is not significantly different from age (FDR corrected *p* ≤ 0.05) based on a DeLong test. Thin dashed lines are variables with significantly different AUCs, all lower than age in (B)–(E) and all higher in (F).

Age outperformed or matched the performance of all other variables as an AD biomarker or clinical stage classifier, except when comparing MCI to AD dementia. Significance patterns were the same regardless of multiple comparison approach. The number of variables whose AUCs were similar to those seen for age increased with progressive AD stage being compared. The percentage of measures tracked by ABC‐DS that performed worse than age at discriminating each AD stage is listed in Table 3, along with the number of variables by type of measure that met or exceeded the AUC seen for age. The superiority of the discriminative ability of age relative to other variables is demonstrated in Figure 2, where the ROC plots for age for each stage comparison (Figure 2A) are plotted against the ROC plots for every other variable (Figure 2B–F). With AD progression, more variables had discriminative abilities similar to age. Only glial fibrillary acidic protein (GFAP), a non‐AD‐specific inflammatory biomarker, matched age for amyloid positivity (Figure 2B), fluid and imaging markers (GFAP, NfL, and pTau‐217, centiloid, volume in key regions like the hippocampus) were useful with tau (Figure 2C), and additional biomarkers and memory measures had AUCs similar to age when comparing the cognitively stable to MCI/cognitive impairment (Figure 2D,E). Age was similar to or outperformed by all variables when differentiating MCI from dementia (Figure 2F), with memory measures and composites including memory significantly outperforming age. AUCs, DeLong values, sample sizes, and thresholds can be found in Tables S2 to S6 for amyloid (2), tau (3), cognitively stable versus impaired (4), cognitively stable versus MCI (5), and MCI versus dementia (6).

**TABLE 3:** Age AUCs relative to other domains: number of domain‐specific variables that met or exceeded age's ability to discriminate AD stage.

Age AUCs relative to other domains					
Stage	Cog/Beh	CSF	Plasma	Imaging	%Worse
Amyloid ‐/+	1	—	1	0	98.5
Tau ‐/+	0	—	3	5	93.8
Stable/MCI	10	—	3	12	79.9
MCI/Dementia	57	—	27	45	0
Stable/Impaired	14	9	5	17	67.3

*Note*: %Worse indicates the percentage of variables whose AUC was significantly worse than that of age. Cog/Beh refers to measures of cognition or behavior. An em dash means the domain is not available for this stage comparison.

The addition of a second variable to age increased AUCs only for the comparison between cognitively stable and impaired. Only short‐term memory tests and summary measures that included memory tests improved group differentiation. These tests included DLD short‐term and cognitive subscale, NTG memory and total composite, DSMSE memory composite, and mCRT total correct. These tests were essential for identifying impairment. The AUCs derived from age and a variable were also significantly higher than just the variable by itself. By combining age and DLD 1 (short‐term memory) together, we were able to reach a maximum AUC of 0.944. The inclusion of *APOE* ε4 status did not increase classification ability for any measure or combination of measures tested for in any stage comparison. AUCs and DeLong values after combining with age and/or *APOE* ε4 status can be found in Tables S2 to S6 for amyloid (2), tau (3), cognitively stable versus impaired (4), cognitively stable versus MCI (5), and MCI versus dementia (6).

## 4 DISCUSSION

The progression of AD in DS, from biological to early cognitive stages, is highly predictable by age alone. Age best differentiates amyloid and tau positivity at roughly the same time (∼41), with age identifying cognitive impairment shortly thereafter (44.5). Age underperformed other variables only when differentiating MCI from dementia. The combination of age and memory‐related cognitive measures improved discrimination, specifically when identifying cognitive impairment.

Age was excellent at classifying individuals by AD biomarker or clinical status in DS except when differentiating MCI from AD dementia. The age–stage consistency is likely due to DS AD resulting from a common genetic cause, leading to a more homogeneous disease progression. This differs from other forms of genetic early‐onset AD, where AD onset and trajectory varies by mutation and is affected by genes.^40^, ^41^ The similar age thresholds for amyloid and tau positivity indicate the two are very tightly coupled, consistent with previous literature.^7^, ^8^ A similar age at amyloid and tau accumulation stands in contrast to late‐onset AD, where tau accumulation begins several years after amyloid.^3^ While the tau age threshold appears to precede amyloid's, the 95% confidence intervals for thresholds put the amyloid threshold as early as 40 and tau as late as 47 years. Age AUCs slightly worsened with clinical status progression and were least discriminative when differentiating MCI from AD dementia. This finding is interesting because it suggests that, while the timing of amyloid and tau positivity is fairly consistent, the variability in later clinical stages is likely influenced by factors other than age.

While the thresholds for age at amyloid and tau positivity are consistent with previous research,^4^, ^7^, ^8^ the thresholds that best differentiated cognitive status in these analyses (44.5) were earlier than previously published estimates (∼52.5years).^1^, ^9^, ^10^ This was likely due to the unbalanced sensitivity and specificity that emerged with the derived threshold. As there is a very low false‐negative and relatively high false‐positive rate for these cognitive thresholds (1.7% and 23.6%, respectively), the early age differentiating cognitive status in these analyses could best be considered a lower bound for when clinicians may start to consider testing for cognitive decline. The previously published estimates may be more representative of an average age at onset for symptomatology rather than when impairment testing should be initiated. The gap between sensitivity and specificity can be visualized in the ROC curves in Figure 2 and exists (to a lesser extent) in the biologically defined stages. A two‐step approach^42^ may be appropriate when staging DS AD, where age is used as an initial screen for individuals with a low probability of PET positivity or cognitive impairment while resources are directed toward confirming AD stage in participants who are more likely to be PET positive or cognitively impaired. The initial high sensitivity thresholds could be combined with the high specificity thresholds in Table 2 to give an age range (e.g., 41.5 to 44.5 for amyloid) where AD stage is most uncertain.

With progressive clinical impairment, several variables were better at differentiating these progressive stages. These included biomarkers with a known connection to AD pathology, with plasma GFAP (AUC 0.878) the measure that emerged earliest as a comparator for age. It is unexpected that this inflammatory biomarker would surpass commonly used markers like the Aβ42/Aβ40 ratio^43^ or newer plasma markers such as pTau‐217 in identifying amyloid accumulation. A recent comparison of pTau‐217 and GFAP found that pTau‐217 increased slightly before GFAP.^44^ ^16^Plasma markers (GFAP, NfL, and pTau‐217) and imaging markers (Centiloid, hippocampal, and precuneus volume) matched age in discriminating tau status. Additionally, biological markers (plasma and imaging markers, tau PET, pTau‐181, subcortical, and temporal‐parietal volumes) and cognitive measures (from the DLD, DSMSE, mCRT, and NTG) were equally effective at identifying clinical impairment. Cognitive measures related to memory were as effective as age in identifying cognitively stable versus impaired individuals and were most useful for distinguishing MCI from AD dementia. The inability of age or biological measures to differentiate MCI from AD dementia might indicate that this distinction is independent of underlying pathology and may be due to challenges in assessing decline within a cohort with existing intellectual disabilities.

Only the combination of age and memory variables (or summary values including memory) significantly increased the AUC over age alone, and only for differentiating cognitively stable individuals from those who are impaired. The highest AUC was 0.944 for the combination of age and DLD 1 (short‐term memory), compared to an AUC of 0.870 for age alone. Combining age with any non‐memory variable, including *APOE* ε4 status, for any other stage did not increase classifier performance. The additive value of considering cognitive measures in identifying cognitive status reflects a large degree of circularity. Both age and cognitive assessment measures, including those used in the analyses, are used explicitly in diagnostic formulation of cognitive impairment, so it is not surprising that individual cognitive measures are associated with cognitive outcomes. The high discriminative power of age and memory in identifying cognitive impairment may ultimately reflect that these variables are essentially predicting themselves.

Our analyses revealed that DS AD stage progression occurred with consistent timing that would allow for early detection and intervention based on age as an early screening tool. This finding is vital as new amyloid antibody treatments may be more effective early in the disease course.^45^ The highly predictable timing of amyloid accumulation in DS would allow for very early interventions; the compressed timeline of AD progression means that anti‐tau medications may need to be given simultaneously with anti‐amyloid drugs. While the high age AUCs suggest a very predictable time course for AD progression, age should not be viewed as a definitive diagnostic marker. Despite the low false‐positive rate in comparing age with amyloid status, it cannot be assumed that all individuals with DS aged 42 years or older have begun accumulating amyloid. Although many people with DS develop dementia, some do not, and a few may never develop amyloid pathology.^46^, ^47^ These individuals could face unnecessary side effects^48^ if treated without confirmed disease status through imaging or fluid tests. Therefore, clinicians should consider starting assessments of amyloid and tau status around age 40 to more accurately identify individuals at risk; this is especially important with the availability of anti‐amyloid and tau therapies. Additionally, establishing a cognitive baseline before age 44 can help detect the onset of cognitive decline more effectively.

These analyses have some limitations. Primary among them is that variable data collection resulted in highly variable sample sizes being compared. As these analyses identified measures with discriminative abilities not significantly different from age, low‐powered analyses are more likely to fail in finding a significant difference. Low power is likely why almost all of the small number of CSF analyses had AUCs not significantly lower than age's, despite values as low as 0.617. In contrast, many of the measures that were not significantly different from age had the largest sample sizes (e.g., DLD, NTG). Additionally, unbalanced sample sizes in the stages being compared may lead to biased AUCs.^49^ We chose not to use the area under the precision‐recall curve because, while it is less sensitive to data imbalance, it does not have DeLong‐like comparison statistics, and its confidence interval comparisons were too conservative. Finally, we cannot discount the possibility of off‐target binding skewing the PET results, particularly as some of the regions used to determine tau positivity were near areas of known off‐target binding, though the partial volume correction used should account for this issue.^50^

Our findings show that AD stage in DS can be predicted by age as accurately as, and sometimes more effectively than, traditional biomarker or cognition‐based methods. The strong link between age and DSAD stage (amyloid positivity, tau positivity, cognitive impairment) offers a crucial opportunity for early detection and timely intervention. Evidence suggests that amyloid and tau start accumulating in the early 40s, highlighting the importance of testing for AD biomarkers in this age range to guide anti‐amyloid and tau treatments. Clinicians should note that detectable decline may appear as early as the mid‐40s, which is earlier than the commonly cited onset in the early 50s. Establishing a cognitive baseline before this potential decline is essential. Combining age with memory measures significantly boosts the ability to identify cognitive decline (AUC = 0.944), though this may reflect circularity. However, only memory measures can distinguish between MCI and dementia. These findings stress the central role of age in guiding screening strategies and clinical trial design while also highlighting the need for memory‐based measures to evaluate cognitive status accurately.

## CONFLICT OF INTEREST STATEMENT

The authors declare no conflicts of interest. Author disclosures are available in the Supporting Information.

## CONSENT STATEMENT

Written informed consent or assent was obtained from all participants or, if the individuals were incapable of providing consent, their legally authorized representatives.

## Supporting information



Supporting Information

Supporting Information

## Data Availability

The dataset supporting the conclusions of this article is available from ABC‐DS at https://abc‐ds.org/researchers/.

## References

[1] Fortea J , Zaman SH , Hartley S , Rafii MS , Head E , Carmona‐Iragui M . Down syndrome‐associated Alzheimer's disease: a genetic form of dementia. Lancet Neurol. 2021;20(11):930. doi:10.1016/S1474-4422(21)00245-3 34687637 PMC9387748

[2] Lott IT , Head E . Dementia in Down syndrome: unique insights for Alzheimer disease research. Nat Rev Neurol. 2019;15(3):135‐147. doi:10.1038/s41582-018-0132-6 30733618 PMC8061428

[3] Jack CR , Knopman DS , Jagust WJ , et al. Tracking pathophysiological processes in Alzheimer's disease: an updated hypothetical model of dynamic biomarkers. Lancet Neurol. 2013;12(2):207‐216. doi:10.1016/S1474-4422(12)70291-0 23332364 PMC3622225

[4] Schworer EK , Zammit MD , Wang J , et al. Timeline to symptomatic Alzheimer's disease in people with Down syndrome as assessed by amyloid‐PET and tau‐PET: a longitudinal cohort study. Lancet Neurol. 2024;23(12):1214‐1224. doi:10.1016/S1474-4422(24)00426-5 39577922 PMC12207984

[5] Day GS . Diagnosing Alzheimer disease. Continuum. 2024;30(6):1584‐1613. doi:10.1212/CON.0000000000001507 39620836

[6] Boerwinkle AH , Gordon BA , Wisch J , et al. Comparison of amyloid burden in individuals with Down syndrome versus autosomal dominant Alzheimer's disease: a cross‐sectional study. Lancet Neurol. 2023;22(1):55‐65. doi:10.1016/S1474-4422(22)00408-2 36517172 PMC9979840

[7] Wisch JK , McKay NS , Boerwinkle AH , et al. Comparison of tau spread in people with Down syndrome versus autosomal‐dominant Alzheimer's disease: a cross‐sectional study. Lancet Neurol. 2024;23(5):500‐510. doi:10.1016/S1474-4422(24)00084-X 38631766 PMC11209765

[8] Zammit MD , Betthauser TJ , McVea AK , et al. Characterizing the emergence of amyloid and tau burden in Down syndrome. Alzheimers Dement. 2024;20(1):388‐398. doi:10.1002/alz.13444 37641577 PMC10843570

[9] Rubenstein E , Tewolde S , Michals A , et al. Alzheimer dementia among individuals with Down syndrome. JAMA Netw Open. 2024;7(9):e2435018. doi:10.1001/jamanetworkopen.2024.35018 39312235 PMC11420697

[10] Sinai A , Mokrysz C , Bernal J , et al. Predictors of age of diagnosis and survival of Alzheimer's disease in Down syndrome. J Alzheimers Dis. 2018;61(2):717‐728. doi:10.3233/JAD-170624 29226868 PMC6004911

[11] Barroeta I , Videla L , Carmona‐Iragui M , Fortea J , Rafii MS . Current advances and unmet needs in Alzheimer's disease trials for individuals with Down syndrome: navigating new therapeutic frontiers. Alzheimers Dement. 2025;21(6):e70258. doi:10.1002/alz.70258 40528298 PMC12173829

[12] Dubois B , Villain N , Schneider L , et al. Alzheimer disease as a clinical‐biological construct—an international working group recommendation. JAMA Neurol. 2024;81(12):1304‐1311. doi:10.1001/jamaneurol.2024.3770 39483064 PMC12010406

[13] Khan T , Khan F , Patil S , et al. Barriers to equitable access: a systematic examination of PET/CT imaging disparities. J Nucl Med. 2025;66(suppl 1):S251597‐S251597.

[14] Leuzy A , Bollack A , Pellegrino D , et al. Considerations in the clinical use of amyloid PET and CSF biomarkers for Alzheimer's disease. Alzheimers Dement. 2025;21(3):e14528. doi:10.1002/alz.14528 40042435 PMC11881640

[15] Nadeau PA , Jobin B , Boller B . Diagnostic sensitivity and specificity of cognitive tests for mild cognitive impairment and Alzheimer's disease in patients with Down syndrome: a systematic review and meta‐analysis1. J Alzheimers Dis. 2023;95(1):13‐51. doi:10.3233/JAD-220991 37522203

[16] Dale AM , Fischl B , Sereno MI . Cortical surface‐based analysis: I. Segmentation and surface reconstruction. Neuroimage. 1999;9(2):179‐194. doi:10.1006/nimg.1998.0395 9931268

[17] Buckner RL , Head D , Parker J , et al. A unified approach for morphometric and functional data analysis in young, old, and demented adults using automated atlas‐based head size normalization: reliability and validation against manual measurement of total intracranial volume. Neuroimage. 2004;23(2):724‐738. doi:10.1016/j.neuroimage.2004.06.018 15488422

[18] Su Y , D'Angelo GM , Vlassenko AG , et al. Quantitative analysis of PiB‐PET with FreeSurfer ROIs. PLoS One. 2013;8(11):e73377. doi:10.1371/journal.pone.0073377 24223109 PMC3819320

[19] Su Y , Blazey TM , Snyder AZ , et al. Partial volume correction in quantitative amyloid imaging. Neuroimage. 2014;107:55. doi:10.1016/j.neuroimage.2014.11.058 25485714 PMC4300252

[20] Su Y , Flores S , Hornbeck RC , et al. Utilizing the centiloid scale in cross‐sectional and longitudinal PiB PET studies. NeuroImage: Clinical. 2018;19:406. doi:10.1016/j.nicl.2018.04.022 30035025 PMC6051499

[21] Mishra S , Gordon BA , Su Y , et al. AV‐1451 PET imaging of tau pathology in preclinical Alzheimer disease: defining a summary measure. Neuroimage. 2017;161:171‐178. doi:10.1016/j.neuroimage.2017.07.050 28756238 PMC5696044

[22] Handen BL , Mapstone M , Hartley S , et al. The Alzheimer's biomarker consortium‐Down syndrome (ABC‐DS): a 10‐year report. Alzheimers Dement. 2025;21(5):e70294. doi:10.1002/alz.70294 40371686 PMC12079517

[23] Handen BL , Lott IT , Christian BT , et al. The Alzheimer's biomarker consortium‐Down syndrome: rationale and methodology. Alzheimers Dement. 2020;12(1):e12065. doi:10.1002/dad2.12065 PMC7396809 32775597

[24] Haxby JV . Neuropsychological evaluation of adults with Down's syndrome: patterns of selective impairment in non‐demented old adults. J Intellect Disabil Res. 1989;33(3):193‐210. doi:10.1111/j.1365-2788.1989.tb01467.x 2526879

[25] Walsh DM , Doran E , Silverman W , Tournay A , Movsesyan N , Lott IT . Rapid assessment of cognitive function in Down syndrome across intellectual level and dementia status. J Intellect Disabil Res. 2015;59(11):1071‐1079. doi:10.1111/jir.12200 26031550 PMC4623954

[26] Gerstadt CL , Hong YJ , Diamond A . The relationship between cognition and action: performance of children 3 1/2‐7 years old on a stroop‐like day‐night test. Cognition. 1994;53(2):129‐153. doi:10.1016/0010-0277(94)90068-x 7805351

[27] Tiffin J , Asher EJ . The purdue pegboard; norms and studies of reliability and validity. J Appl Psychol. 1948;32(3):234‐247. doi:10.1037/h0061266 18867059

[28] McCarthy DA . Manual for the McCarthy Scales of Children's Abilities. Psychological Corporation; 1972. . Accessed September 4, 2025. https://cir.nii.ac.jp/crid/1971149384766648244

[29] Devenny DA , Zimmerli EJ , Kittler P , SJ Krinsky‐McHale . Cued recall in early‐stage dementia in adults with Down's syndrome. J Intellect Disabil Res. 2002;46(Pt 6):472‐483. doi:10.1046/j.1365-2788.2002.00417.x 12354318

[30] Beery KE , The Beery‐Buktenica developmental test of visual‐motor integration. Administration Scoring and Teaching Manual . 1997. Accessed September 4, 2025. https://cir.nii.ac.jp/crid/1571980075429864064

[31] Wechsler D . Wechsler Intelligence Scale for Children. Psychological Corporation; 1949.

[32] Tinetti ME . Performance‐oriented assessment of mobility problems in elderly patients. J Am Geriatr Soc. 1986;34(2):119‐126. doi:10.1111/j.1532‐5415.1986.tb05480.x 3944402 10.1111/j.1532-5415.1986.tb05480.x

[33] Evenhuis HM . The dementia questionnaire for people with learning disabilities. In: Prasher VP , ed. Neuropsychological Assessments of Dementia in Down Syndrome and Intellectual Disabilities. Springer International Publishing; 2018:43‐56. doi:10.1007/978‐3‐319‐61720‐6_3

[34] Esralew L , Janicki MP , Keller SM . National task group early detection screen for dementia (NTG‐EDSD). In: Prasher VP , ed. Neuropsychological Assessments of Dementia in Down Syndrome and Intellectual Disabilities. Springer International Publishing; 2018:197‐213. doi:10.1007/978‐3‐319‐61720‐6_11

[35] Reiss S . Reiss Screen for Maladaptive Behavior. IDS Publishing Corporation; 1988.

[36] Hosmer DW Jr , Lemeshow S , Sturdivant RX . Assessing the fit of the model. Applied Logistic Regression. John Wiley & Sons Ltd; 2013:153‐225. doi:10.1002/9781118548387.ch5

[37] Youden WJ . Index for rating diagnostic tests. Cancer. 1950;3(1):32‐35. doi:10.1002/1097‐0142(1950)3:1<32::aid‐cncr2820030106>3.0.co;2‐3 15405679 10.1002/1097-0142(1950)3:1<32::aid-cncr2820030106>3.0.co;2-3

[38] Robin X , Turck N , Hainard A , et al. pROC: an open‐source package for R and S+ to analyze and compare ROC curves. BMC Bioinf. 2011;12(1):77. doi:10.1186/1471‐2105‐12‐77 10.1186/1471-2105-12-77 PMC3068975 21414208

[39] DeLong ER , DeLong DM , DL Clarke‐Pearson . Comparing the areas under two or more correlated receiver operating characteristic curves: a nonparametric approach. Biometrics. 1988;44(3):837‐845. 3203132

[40] Daniels AJ , McDade E , Llibre‐Guerra JJ , et al. 15 years of longitudinal genetic, clinical, cognitive, imaging, and biochemical measures in DIAN. medRxiv . 2024;2024.08.08.24311689. Published 2024 Aug 9. doi:10.1101/2024.08.08.24311689 10.1038/s44400-025-00047-7 PMC12909123 41709913

[41] Chhatwal JP , Schultz SA , McDade E , et al. Variant‐dependent heterogeneity in amyloid β burden in autosomal dominant Alzheimer's disease: cross‐sectional and longitudinal analyses of an observational study. Lancet Neurol. 2022;21(2):140‐152. doi:10.1016/S1474‐4422(21)00375‐6 35065037 10.1016/S1474-4422(21)00375-6 PMC8956209

[42] Brum WS , Cullen NC , Janelidze S , et al. A two‐step workflow based on plasma p‐tau217 to screen for amyloid β positivity with further confirmatory testing only in uncertain cases. Nat Aging. 2023;3(9):1079‐1090. doi:10.1038/s43587‐023‐00471‐5 37653254 10.1038/s43587-023-00471-5 PMC10501903

[43] Zhou Y , Sheehan R , Guo L , Strydom A . Blood‐based biomarkers for Alzheimer's disease in Down syndrome: a systematic review and meta‐analysis. Alzheimers Dement. 2025;21(4):e70135. doi:10.1002/alz.70135 40219863 10.1002/alz.70135 PMC11992652

[44] Boerwinkle AH , Wisch JK , Handen BL , et al. The mediating role of plasma glial fibrillary acidic protein in amyloid and tau pathology in Down's syndrome. Alzheimers Dement. 2025;21(1):e14359. doi:10.1002/alz.14359 39535359 10.1002/alz.14359 PMC11782213

[45] Sims JR , Zimmer JA , Evans CD , et al. Donanemab in early symptomatic Alzheimer disease: the TRAILBLAZER‐ALZ 2 randomized clinical trial. JAMA, J Am Med Assoc. 2023;330(6):512‐527. doi:10.1001/jama.2023.13239 10.1001/jama.2023.13239 PMC10352931 37459141

[46] Liou JJ , Lou J , Flores‐Aguilar L , et al. A neuropathology case report of a woman with Down syndrome who remained cognitively stable: implications for resilience to neuropathology. Alzheimers Dement. 2025;21(2):e14479. doi:10.1002/alz.14479 39868632 10.1002/alz.14479 PMC11851131

[47] Doran E , Keator D , Head E , et al. Down syndrome, partial trisomy 21, and absence of Alzheimer's disease: the role of APP. J Alzheimers Dis. 2017;56(2):459‐470. doi:10.3233/JAD‐160836 27983553 10.3233/JAD-160836 PMC5662115

[48] Sperling RA , Jack CR , Black SE , et al. Amyloid related imaging abnormalities (ARIA) in amyloid modifying therapeutic trials: recommendations from the Alzheimer's association research roundtable workgroup. Alzheimers Dement. 2011;7(4):367‐385. doi:10.1016/j.jalz.2011.05.2351 21784348 10.1016/j.jalz.2011.05.2351 PMC3693547

[49] Movahedi F , Padman R , Antaki JF . Limitations of receiver operating characteristic curve on imbalanced data: assist device mortality risk scores. J Thorac Cardiovasc Surg. 2023;165(4):1433‐1442.e2. doi:10.1016/j.jtcvs.2021.07.041 34446286 10.1016/j.jtcvs.2021.07.041 PMC8800945

[50] Flores S , Chen CD , Su Y , et al. Investigating tau and amyloid tracer skull binding in studies of Alzheimer disease. J Nucl Med. 2023;64(2):287‐293. doi:10.2967/jnumed.122.263948 35953305 10.2967/jnumed.122.263948 PMC9902848

